# Pathology in the artificial intelligence era: Guiding innovation and implementation to preserve human insight

**DOI:** 10.1016/j.acpath.2025.100166

**Published:** 2025-02-28

**Authors:** Harry Gaffney, Kamran M. Mirza

**Affiliations:** a Concord Clinical School, Faculty of Medicine and Health, The University of Sydney, Sydney, New South Wales, Australia; b The Godfrey D. Stobbe Professor of Pathology Education, Assistant Chair for Education and Director of the Division of Training, Programs and Communication, University of Michigan (Michigan Medicine) Department of Pathology, Ann Arbor, MI, USA

**Keywords:** AI-Assisted diagnostics, Algorithmic transparency, Artificial intelligence, Diagnostic accuracy, Diagnostic augmentation, Educational frameworks, Governance, Pathology, Public engagement, Workflow optimization

## Abstract

The integration of artificial intelligence in pathology has ignited discussions about the role of technology in diagnostics—whether artificial intelligence serves as a tool for augmentation or risks replacing human expertise. This manuscript explores artificial intelligence's evolving contributions to pathology, emphasizing its potential capacity to enhance, rather than eclipse, the pathologist's role. Through historical comparisons, such as the transition from analog to digital in radiology, this paper highlights how technological advancements have historically expanded professional capabilities without diminishing the essential human element. Current applications of artificial intelligence in pathology—from diagnostic standardization to workflow efficiency—demonstrate its potential to augment diagnostic accuracy, expedite processes, and improve consistency across institutions. However, challenges remain in algorithmic bias, regulatory oversight, and maintaining interpretive skills among pathologists. The discussion underscores the importance of comprehensive governance frameworks, evolving educational curricula, and public engagement initiatives to ensure artificial intelligence in pathology remains a collaborative endeavor that empowers professionals, upholds ethical standards, and enhances patient outcomes. This manuscript ultimately advocates for a balanced approach where artificial intelligence and human expertise work in concert to advance the future of diagnostic medicine.

## Introduction

The integration of artificial intelligence (AI) into pathology presents a powerful opportunity to enhance diagnostic accuracy, efficiency, and consistency.^1^ Unlike some past technological advancements that risked displacing human roles, AI in pathology has the potential to function as an augmentative tool to support rather than replace the expertise of pathologists.^1^^,^^2^ This manuscript explores AI's role through historical parallels—such as the transition to digital imaging in radiology and the adoption of microscopes in pathology—that demonstrate how technology has historically expanded, rather than reduced, professional capacities.^2^ By examining specific applications of AI in pathology, from complex pattern recognition to workflow optimization, we highlight how AI serves as a valuable ally in diagnostics.^3^ Ultimately, the responsible integration of AI into pathology depends on maintaining a balance between technological innovation and the irreplaceable judgment of skilled professionals. This balance requires that AI algorithms remain robust, supervised, and constrained to prevent systems from self-adjusting thresholds for human review without oversight. Ensuring that pathologists retain their interpretive role is essential for upholding patient safety and professional accountability.^4^, ^5^, ^6^ Artificial intelligence's role in pathology could extend across a range of applications, including enhancing diagnostic accuracy in complex cases, streamlining routine workflows, and standardizing practices across institutions.^1^^,^^7^^,^^8^ These applications are not only technical advancements but also touch on critical aspects of governance, ethical oversight, and educational preparation.^9^, ^10^, ^11^ This manuscript explores how AI integration in pathology fits within these broader frameworks, addressing the regulatory structures required to guide its responsible use, the essential training needed to support pathologists in a digital era, and the practical impact of AI on daily diagnostic practices. Through this lens, we examine how AI serves as an augmentative tool, reinforcing rather than replacing the expertise and judgment of pathologists.

## Terminology and key concepts

In this manuscript, the terms “AI augmentation” and “AI-assisted diagnostics” are used to describe how AI has the potential to support and enhance, rather than replace, the expertise of pathologists.^2^ “AI augmentation” refers to the role of AI technologies in expanding the pathologist's capabilities, such as identifying complex patterns that may aid diagnostic accuracy.^12^ “AI-assisted diagnostics” highlights applications where AI tools support specific diagnostic tasks, like automating routine screenings or suggesting potential anomalies for further review.^13^^,^^14^ Both terms emphasize AI's function as a complementary asset within pathology, enhancing efficiency and precision while maintaining the crucial role of human expertise in clinical decision-making.

## Historical technology: adoption and the augmentation of expertise

Throughout history, technological advancements have frequently supported and augmented professional expertise rather than replacing it.^15^, ^16^, ^17^ In pathology, tools like the microscope revolutionized the field by allowing pathologists to view cellular structures with unprecedented detail, yet these tools still required the pathologist's interpretive skills to derive meaning from these observations.^18^ Similarly, the development of the automobile transformed transportation by improving efficiency and accessibility. However, skilled drivers remained essential for safe travel as technology alone could not replace human decision-making.^19^ The same evolution occurred in radiology, where the shift from analog to digital imaging enhanced diagnostic accuracy and efficiency without replacing the radiologist's critical role in interpretation and clinical judgment.^20^ Another relevant example is the widespread adoption of calculators in scientific research. While these tools sped up calculations and reduced human error in numerical tasks, scientists and researchers still needed their expertise to interpret the data, design experiments, and draw conclusions.^21^

However, as AI evolves, its capacity to mimic human decision-making—such as in automated trading systems in financial markets—raises the possibility of technology taking over functions traditionally held by humans.^22^ These systems, capable of analyzing vast datasets and making rapid decisions without human intervention, have already transformed industries by outperforming human traders in speed and precision,^23^ yet even in financial markets, human oversight remains critical to address ethical considerations, manage risks, and provide context to algorithmic decisions, particularly in complex and unpredictable market conditions.^24^ Similarly, in pathology, while AI has the potential to handle repetitive tasks and even assist in decision-making, its role should remain augmentative rather than completely replace human expertise.^25^^,^^26^ Pathologists must still guide AI, providing oversight in nuanced cases, particularly those requiring clinical context, empathy, and complex judgment.^27^^,^^28^

## Historical technology: pathology adoption vs AI integration

Pathology has historically embraced technological innovations, from adopting techniques such as immunohistochemistry, flow cytometry phenotyping, cytogenetics, and polymerase chain reaction (PCR) to integrating digital imaging systems.^1^^,^^29^, ^30^, ^31^ These advancements have significantly enhanced diagnostic and prognostic accuracy, increased the breadth of detectable anomalies, and streamlined laboratory operations.^30^^,^^32^^,^^33^ For instance, digital pathology has allowed for the remote viewing and diagnosis of slides, revolutionizing the speed at which second opinions can be gathered and facilitating more collaborative diagnostic processes.^2^^,^^34^

Today, AI appears poised to redefine the role of technology in pathology.^35^ Unlike prior innovations, AI offers assistance and, in some cases, the potential to replace specific routine tasks previously overseen by pathologists.^36^ For example, AI is already capable of performing high-throughput, repetitive tasks such as pre-screening of hematology blood smears.^32^^,^^37^, ^38^, ^39^ These systems can efficiently identify cases needing further review, potentially reducing the pathologist's direct involvement in the initial stages of routine diagnostics.^40^ However, complex diagnostic tasks that require nuanced interpretation and contextual analysis remain firmly in the realm of human expertise.^2^ While AI may take on more routine tasks and alleviate workloads in high-volume screenings, it must remain subject to robust human oversight to ensure that critical cases requiring expert review are not bypassed.^41^ Constraining AI's ability to autonomously adjust thresholds for human review is crucial; only pathologists should control such parameters to prevent unintended removal from key decision-making processes.

Nonetheless, AI's capabilities for analyzing extensive datasets and identifying intricate patterns could promise substantial support in diagnostic processes.^42^^,^^43^ In particular, AI's proficiency in handling routine and repetitive tasks autonomously has sparked debates about the future role of pathologists as it introduces the potential to replace certain preliminary stages of routine diagnostics.^43^ However, the complex interpretive functions of pathology still require human expertise as AI's current potential capabilities are primarily suited to assist pathologists by reducing routine workload rather than replacing them entirely.^44^

## Enhancement vs replacement: augmenting capabilities and potential for replacement

Artificial intelligence's current utilization underscores its role as an enhancer. In complex histopathological analysis and predictive analytics, AI algorithms offer speed and accuracy that complement the depth of a pathologist's expertise, allowing more time for complex cases and reducing routine workload.^43^ By automating routine processes, such as initial screenings and pattern recognition in straightforward cases, AI enables pathologists to prioritize cases requiring specialized interpretive skills.^45^

While AI assists with routine tasks, it is essential to maintain a structured approach to training that ensures pathologists develop a comprehensive skill set.^1^^,^^42^^,^^46^ One approach involves hybrid workflows, where junior pathologists initially review cases with AI support but also independently handle diverse and complex cases. This balanced exposure fosters diagnostic acumen across both routine and challenging cases. Additionally, mentorship programs, where seasoned pathologists review cases with trainees, further reinforce skill development.

For instance, AI software like Paige.AI flags potential cancerous lesions in digital slides, allowing pathologists to focus on nuanced tasks such as tumor grading and staging.^47^^,^^48^ Such practices ensure that while AI streamlines routine tasks, pathologists retain direct engagement with varied case complexities necessary for skill enhancement.

Conversely, AI's ability to automate processes presents a scenario where certain tasks may no longer require a pathologist's direct oversight. Routine screenings, such as those for cervical cytology, have become targets for automation, suggesting a future where AI's scope could expand to screening and potentially more comprehensive diagnostic roles.^48^ Automated systems are already used in pre-screening cervical cytology slides and automated hematology analyzers for blood smears, flagging cases for further review and effectively reducing the workload for pathologists and medical laboratory scientists.^49^^,^^50^

## The power of control in influencing replacement vs augmentation of AI within health care

The path AI takes in health care, particularly in pathology, hinges on a crucial factor: control. As economist Daron Acemoglu argues in *Power and Progress* and other works, the impact and trajectory of any new technology are shaped significantly by whether it is directed by those with firsthand knowledge of the field or by concentrated corporate interests.^51^, ^52^, ^53^, ^54^ When technology development is guided by practitioners who understand the daily complexities of their field, innovations tend to be applied in ways that augment and enhance professional capabilities, fostering collaboration and advancing the profession and society at large.^51^^,^^52^^,^^54^ However, when powerful corporations primarily control technological advancements, driven by profit rather than practice, the risk of replacement rather than augmentation increases.^51^^,^^52^^,^^54^ Such control may lead to diminished roles for human expertise and the broader societal benefits that could be achieved may be frequently limited as a result.^51^^,^^52^^,^^54^

In pathology, the implications of control over AI development are especially consequential. When pathologists and healthcare practitioners themselves lead AI integration, the potential for technology is more likely to complement, rather than compete with, the nuanced expertise pathologists bring to each case.^55^, ^56^, ^57^ Current pathologist-led initiatives illustrate this collaborative approach, underscoring the value of involving domain experts in AI development for meaningful augmentation. One example is RudolfV, a foundation model in computational pathology developed through significant collaboration with pathologists.^58^^,^^59^ This model, created with input from more than 15 laboratories and crafted to meet clinical needs, excels in tasks such as tumor microenvironment profiling and biomarker evaluation, areas that benefit from the unique insights and data pathologists provided.^58^^,^^59^ By prioritizing the interpretative and complex requirements of pathology, RudolfV has not only set a high standard for diagnostic accuracy and robustness but also demonstrated how pathologist-driven AI can be tailored specifically to enhance and streamline the diagnostic process without displacing essential human oversight.^58^^,^^59^

Similarly, the Ecosystem for Pathology Diagnostics with AI Assistance (EMPAIA) initiative is a notable example of collaborative, pathologist-led AI development.^60^, ^61^, ^62^, ^63^ Ecosystem for Pathology Diagnostics with AI Assistance operates as an open, vendor-neutral platform that brings together pathologists, computer scientists, and industry stakeholders to address challenges in integrating AI into daily pathology practice.^60^, ^61^, ^62^, ^63^ By establishing interoperability standards and guidelines for AI testing, as well as methods for enhancing the transparency of AI decision-making, EMPAIA ensures that various AI-based image analysis applications from different vendors can function seamlessly within the same diagnostic framework.^60^, ^61^, ^62^, ^63^ This initiative showcases the critical role pathologists play in shaping AI development to meet practical clinical needs, prioritizing flexibility, reliability, and ethical standards. Through such standardization, EMPAIA highlights how pathologist-led AI applications can ensure clinical relevance, fostering a collaborative approach that integrates AI tools into diagnostic workflows without overshadowing human expertise.

Another significant example is NaviPath, a human-AI collaborative navigation system that exemplifies how AI can augment rather than replace pathologists.^64^ This system was designed specifically to aid pathologists in navigating high-resolution tumor images, making it easier and faster to identify pathological patterns.^64^ In evaluations, pathologists using NaviPath could detect more than twice as many relevant patterns compared to manual navigation, achieving higher precision and recall.^64^ Such tools empower pathologists by enhancing efficiency without replacing the critical interpretative skills required for accurate diagnostics. NaviPath illustrates the potential for AI to act as a supportive tool that complements a pathologist's expertise, particularly in tasks that demand both technical precision and clinical context.

Historically, we see a similar dichotomy in technology deployment, where control over innovations determined whether technologies supported or supplanted professional roles. During the industrial revolution, for instance, the application of steam engines—largely driven by skilled craftspeople—resulted in productivity gains that empowered skilled workers, fostering cycles of innovation and advancement across industries.^65^ In contrast, the mechanization of the textile industry, controlled by a small number of industrialists, led to the replacement of skilled artisans with low wage, often child laborers, fueling social unrest and the Luddite revolt.^65^ This contrast highlights how the democratization of technology tends to support workers by enhancing their capabilities, while concentrated corporate control can erode skilled roles and limit broad societal benefits.

For pathology, the lesson from history is clear: ensuring that AI remains a tool for augmentation will require concerted efforts to involve pathologists directly in AI development and oversight.^66^^,^^67^ This could include establishing pathologist-led advisory boards to oversee AI applications, forming dedicated research partnerships with AI developers to ensure that patient outcomes and clinical needs are prioritized and creating continuous feedback channels that allow pathologists to adapt AI systems according to evolving clinical and ethical considerations.^68^^,^^69^ Such structures enable pathologists to guide the integration of AI in ways that align with the profession's standards and uphold patient care.^70^ Only with such intentional, pathologist-led frameworks can AI continue to serve as an empowering tool that complements the diagnostic process, expands professional capabilities, and enhances patient care—rather than becoming a disruptive force that risks diminishing the essential interpretative and contextual role of human expertise in pathology.^69^

## Potential risks of AI in pathology

While AI offers numerous potential advantages in pathology, it is essential to approach its integration critically to prevent over-reliance and potential complacency.^71^^,^^72^ One significant risk is that excessive dependence on AI for routine and repetitive tasks could lead to the de-skilling of pathologists, particularly in areas that require interpretive precision.^73^ If diagnostic workflows become dominated by AI systems, pathologists might lose familiarity with essential skills, especially those needed for complex, nuanced interpretations.^74^

To mitigate this, AI systems must be carefully controlled to ensure that they cannot autonomously revise thresholds for human review. If algorithms are allowed to “decide" that their assessments surpass the need for human oversight, as cautioned in Nexus, there is a risk that cases requiring professional scrutiny could bypass pathologist review altogether.^75^ By ensuring that only human experts control diagnostic thresholds, the profession may preserve interpretive expertise and uphold patient care standards. This dependency could also ultimately reduce the pathologist’s autonomy, limiting their ability to provide independent analysis without AI support.^76^ Therefore, it is essential that pathologists remain actively engaged in diagnostic tasks—particularly those that demand critical human insight—and retain control over decision-making thresholds to reinforce accountability and protect the integrity of diagnostic workflows.

A related concern is the influence of corporate interests on AI development, where efficiency and cost reduction may be prioritized over diagnostic quality.^77^^,^^78^ When AI applications are controlled by corporate entities outside the healthcare sector, the focus often shifts toward automating high-volume, lower-complexity tasks to reduce labor costs.^79^^,^^80^ While such efficiencies might benefit corporate stakeholders, they may risk eroding the professional roles of pathologists, potentially transferring control from healthcare professionals to corporations over time.^80^ To safeguard the integrity of the pathology profession, advocacy within the community is needed to guide AI development toward augmenting, rather than replacing, the work of pathologists.

To ensure AI serves as a complementary tool in pathology, several practical steps are recommended. First, pathologists should engage in governance frameworks within healthcare institutions, advocating for standards that ensure AI is implemented ethically and responsibly.^81^ These frameworks should address essential areas like data privacy, algorithm transparency, and accountability to mitigate risks.^82^ Additionally, continuous education is crucial; by staying informed about AI advancements, pathologists can better understand the strengths and limitations of AI tools, allowing them to apply critical oversight in their practice.^81^ Finally, fostering collaboration between pathologists and AI developers can help ensure that AI tools align with clinical needs, enhancing diagnostic capabilities rather than replacing them.^83^

Overall, while AI has transformative potential in pathology, careful oversight is essential to ensure it remains an augmentative tool rather than a replacement. Maintaining human control over diagnostic thresholds is critical to preserving interpretive expertise and safeguarding patient care standards. By actively engaging in advocacy, fostering collaboration with AI developers, and prioritizing ongoing education, pathologists can uphold their central role in patient care while integrating AI responsibly to enhance diagnostic capabilities.

Through a combination of advocacy, collaboration, and education, pathologists can maintain their central role in patient care while exploring responsible integration the benefits of AI into their field.

## Enhancing speed and efficiency while preserving depth of insight

Artificial intelligence's potential for impact on operational efficiency is undeniable. With the ability to process data and images at unprecedented speeds, AI can dramatically reduce the turnaround times for test results, directly benefiting patient care through quicker diagnostics. An example of this is using AI to rapidly review breast biopsy samples, where algorithms can scan slides and prioritize those with potential malignancies for earlier review by a pathologist.^84^^,^^85^

Despite these advances, the nuanced insights offered by pathologists remain vital. The depth of diagnostic insight, encompassing prognostic information and complex case interpretations, continues to rely heavily on human expertise, underscoring AI's complementary rather than substitutive role in pathology.^86^

## Error reduction and new challenges

Artificial intelligence technologies have the potential to enhance the accuracy and consistency of pathological assessments, reducing human errors that can occur due to fatigue, high workload, or subjective interpretation.^87^ Artificial intelligence excels in performing quantitative tasks with high precision, such as measuring tumor sizes or counting cell populations.^88^ For example, AI algorithms can process images and provide measurements with a level of consistency that is challenging for humans to maintain, particularly over extended periods.^89^

Artificial intelligence also contributes significantly to the standardization of diagnostics by helping to standardize the interpretation of pathological samples across different institutions. In pathology, variability in diagnostic interpretations can arise from differences in training backgrounds, subjective interpretation, and institutional resources, which, as research indicates, can impact diagnostic consistency and, consequently, patient outcomes.^90^ This is particularly critical in complex cases, where diagnostic interpretations may vary based on individual pathologists' experiences and expertise.^91^^,^^92^ By analyzing diverse datasets and learning from a broad range of cases, AI can help mitigate these discrepancies, offering consistent diagnostic suggestions.^93^ For example, AI applications have shown promise in reducing variability in tumor grading and classification across institutions by applying uniform criteria for diagnostic interpretation.^94^ This approach is especially valuable in less specialized centers, where specific expertise may be limited, thereby supporting diagnostic equity and improving standardization in healthcare settings.^95^

While AI offers these significant advantages, it also introduces challenges that must be addressed to ensure its safe and effective implementation in pathology practices. One such challenge is potential algorithmic bias.^96^ Because AI systems learn from data, they can inherit and perpetuate biases present in training datasets.^97^ For example, an AI model trained predominantly on data from a specific demographic may underperform when applied to a broader, more diverse population, potentially resulting in disparities in diagnostic accuracy and healthcare outcomes.^98^ To mitigate these risks, it is essential to implement multiple strategies. Ensuring that training datasets are diverse and representative of varied demographic, geographical, and socioeconomic backgrounds is one primary method to reduce potential bias.^99^ Additionally, continuous validation and periodic audits can help identify performance discrepancies across different demographic groups when the AI model is tested in real-world settings.^100^ For instance, routine performance checks may reveal tendencies to overdiagnose or underdiagnose certain subgroups, prompting necessary adjustments in the model's parameters or training data.^101^

Incorporating feedback loops is also potentially beneficial, as they may allow AI systems to learn continually from new data, improving accuracy, and may enable the detection of emerging biases as algorithms encounter broader patient data.^101^ Establishing protocols for algorithmic adjustments is equally important as these protocols should outline steps for recalibrating AI models based on demographic shifts or healthcare trends.^102^ Such measures help ensure that AI algorithms evolve in alignment with population changes and maintain diagnostic consistency across diverse patient groups.

Another limitation of AI lies in its handling of complex cases, where its inability to fully understand clinical context may become apparent. While AI can identify patterns and anomalies, interpreting these findings within a patient's overall clinical picture often requires human insight.^103^ For instance, AI might detect abnormal cell structures but may not effectively correlate these with other critical clinical symptoms or test results, underscoring the continued importance of human expertise in complex diagnostics.^104^

For new pathologists, gaining the necessary expertise to interpret such cases independently is crucial. Structured mentorship and supervised training play essential roles in this phase, helping junior pathologists navigate complex cases with confidence.^35^

In hybrid AI-human workflows, junior pathologists can initially review cases with AI support while engaging independently with diverse and challenging cases.^105^ This approach balances reliance on AI with the development of diagnostic acumen and interpretive skills, particularly where clinical context is essential. Mentorship from seasoned pathologists allows trainees to review cases, discuss discrepancies, and refine their diagnostic judgment, ensuring they develop the experience required for nuanced cases.^106^ By ensuring that only human experts can revise the thresholds for case prioritization, this supervised training enhances their ability to make informed decisions when AI outputs may be insufficient or conflicting, safeguarding patient outcomes through robust clinical judgment and preserving the pathologist's essential role in diagnostic workflows.

Security and data privacy are significant concerns as well, as pathology AI systems, like other digital technologies, may be vulnerable to cybersecurity risks.^107^ Notably, the 2016 collaboration between Google's DeepMind and the Royal Free London National Health Service (NHS) Trust for kidney disease prediction violated data protection laws by sharing patient data without proper consent, underscoring the need for transparency and compliance in data handling.^108^ Incidents such as this highlight the importance of stringent cybersecurity measures and regulatory compliance to protect patient data and maintain trust in AI applications within health care.

Finally, the rapid advancement of AI in health care poses potential challenges for regulatory bodies tasked with ensuring the safety and efficacy of these tools.^109^ Developing standards and guidelines that keep pace with AI innovation is essential to protect patients and ensure that AI tools are used responsibly and effectively within healthcare systems.^110^ Existing standards, such as the Food and Drug Administration (FDA) guidelines on AI and machine learning for medical devices, and recommendations by the World Health Organization (WHO) on digital health technologies provide a foundational regulatory framework for AI in health care.^110^ These guidelines emphasize transparency, algorithmic accountability, and patient safety. However, as AI capabilities evolve, these frameworks may require regular updates to address emerging complexities in AI applications.

For instance, while current FDA guidelines outline general safety and performance standards, they require that AI algorithms remain “locked” post approval, preventing any modification.^111^ While this policy ensures model stability and guards against potentially harmful updates, it can lead to performance decay over time, especially in the rapidly evolving healthcare landscape.^111^ Shifts in patient demographics, clinical practices, and treatment modalities may all contribute to a decline in model accuracy.^111^ Consequently, regulatory bodies must consider protocols for periodic reassessment and revalidation of AI tools. Additionally, harmonizing these standards on an international scale, potentially through organizations like the WHO, could support equitable and consistent AI deployment across global healthcare systems, addressing variations in regulatory rigor between regions.

## Adapting educational pathways for AI integration in pathology

As AI may become integral to pathology, educational programs must adapt to prepare pathologists with the necessary skills for this evolving field.^35^ Traditional curricula, which emphasize morphological analysis and laboratory management, now may require the inclusion of core AI competencies, such as data science fundamentals, ethical considerations, and algorithmic transparency.^112^

Professional organizations and institutions are beginning to incorporate AI into their training frameworks.^113^ Programs like the Pathology Informatics Essentials for Residents introduce modules on machine learning principles and digital pathology tools, providing residents with hands-on experience in AI-assisted diagnostics.^114^ Other institutions, such as Stanford and the Mayo Clinic, have developed AI-focused workshops, enabling pathologists to interpret AI outputs and understand ethical applications in clinical practice.^115^^,^^116^ To support continuous education for practicing pathologists, professional development courses emphasize critical areas like AI ethics, algorithmic transparency, and the practical use of AI in diagnostics. These courses help ensure that pathologists can effectively evaluate and integrate AI tools while maintaining their diagnostic integrity and clinical judgment.

## The path forward

### Envisioning a symbiotic future: optimal collaboration between AI and pathologists

As we look toward the potential future of pathology with the integration of AI, envisioning a symbiotic relationship between AI and pathologists becomes imperative. This partnership aims to harness the strengths of both human expertise and AI's potential capabilities to advance the field in previously unimaginable ways.

In this symbiotic future AI would, with appropriate human oversight to prevent shifts in human-review thresholds, ideally handle the high-volume, repetitive tasks that are essential yet time-consuming for pathologists. This includes tasks like the initial screening of slides, quantification tasks such as counting mitotic figures, and preliminary data analysis, which can be labor-intensive. By automating these processes, AI may free pathologists to focus on more complex diagnostic decisions, interpretative tasks, and personalized patient care strategies.^67^

For example, in breast cancer cases, AI can quickly sift through thousands of histology slides to detect and prioritize abnormal cases, presenting only those needing further review.^117^ Pathologists would then apply their expertise to these selected cases, focusing on nuanced aspects like tumor grading and staging, which are critical for determining the appropriate treatment plans. Additionally, as pathology practices evolve to become more patient-centered, this shift could be ideal for freeing up pathologists' time to meet directly with patients. This could allow pathologists to provide direct patient consultations, which may improve patient understanding of diagnostic findings and facilitate rapid follow-up on clinical queries. This setup not only supports personalized patient care but also enables clinicians and pathologists to collaborate closely, ensuring that diagnostic insights are aligned with treatment plans while further empowering patients. By establishing a space for these direct interactions, pathology clinics bridge the gap between diagnostics and patient care, enhancing the role of the pathologist as a clinical consultant.

### Policy and governance

Effective governance and ethical frameworks are critical to guiding the responsible development and use of AI in pathology. Artificial intelligence introduces potential unique challenges to current regulatory frameworks due to its reliance on vast datasets, evolving algorithms, and the need for transparency in decision-making. While existing standards, such as those from the FDA for medical devices or the WHO's ethical guidelines on AI in health care, provide a regulatory foundation, they may fall short in addressing AI's rapid advancements and the complexities of diagnostic applications.^110^ For example, current standards often lack specific requirements for algorithm transparency or periodic audits to ensure ongoing reliability across diverse patient demographics.^110^

These gaps may directly impact pathologists' daily work, as unclear or inconsistent standards could expose institutions to liability risks or lead to variability in diagnostic quality. Multi-level policies are therefore essential, with institutional policies providing immediate oversight, national standards ensuring baseline safety and efficacy, and international guidelines, such as WHO classifications, fostering consistency across borders.^110^ Such frameworks could help ensure that AI tools support high-quality patient care without compromising the pathologist's role or ethical standards.^110^

Governance structures are likely needed at multiple levels—local/institutional, national, and international—to create a cohesive and ethically sound framework that supports AI integration while ensuring equitable and transparent practices.^118^ These governance structures are not only theoretical but directly impact the day-to-day work of pathologists. Adhering to standardized protocols and undergoing regular training on AI compliance, ethics, and the limitations of AI tools is essential for ensuring that pathologists can integrate these technologies responsibly within their clinical practice.^81^ Institutions must ensure that pathologists and laboratory staff members are regularly updated on evolving AI regulations at the institutional, national, and global levels. Such continuous education may align practice with established guidelines and help ensure that pathologists remain proficient in using AI tools while maintaining their critical oversight role in diagnostics.

#### Local and institutional levels

Hospitals and pathology departments play a key role by establishing oversight policies tailored to their specific workflows and patient populations.^119^ This could include implementing AI performance audits that evaluate the accuracy, consistency, and relevance of AI outputs in real-world cases, ensuring that these tools continue to meet clinical standards.^120^ Quality-control protocols would further monitor AI systems to verify that they perform consistently over time, particularly in handling sensitive data and complex cases.^111^ Additionally, institutions could provide ongoing training programs for pathologists and laboratory staff, focusing on responsible AI use, ethical considerations, and understanding the limitations of AI models in the context of pathology.

#### National level

Professional organizations, such as the College of American Pathologists or the Royal College of Pathologists (RCPath), could develop comprehensive standards that set a regulatory baseline for AI in pathology. These standards would cover crucial areas such as algorithmic transparency, ensuring that AI tools used in pathology are explainable and interpretable to clinicians. They could also establish guidelines around accountability, defining the legal and ethical responsibilities of AI developers, institutions, and practitioners in the event of diagnostic discrepancies. National standards could further address patient data privacy, reinforcing compliance with data protection laws like Health Insurance Portability and Accountability Act (HIPPA) in the United States or General Data Protection Regulation (GDPR) in the European Union. By creating a consistent regulatory framework, these professional bodies may support the uniform and safe application of AI across institutions nationwide.

#### International level

Organizations like the WHO could play a critical role in setting global guidelines that promote consistency in AI applications across borders.^121^ Such guidelines would aim to harmonize AI standards, ensuring that AI tools used in pathology adhere to universal principles of ethical use, transparency, and patient safety.^121^ This international oversight is particularly important in regions with fewer resources or limited healthcare infrastructure, where adopting consistent standards could help reduce disparities in access to quality AI-driven diagnostic tools. Additionally, the WHO could provide resources and support for nations looking to develop their own AI regulations, fostering a cooperative global approach that respects regional differences while upholding core standards. Cross-border alignment through WHO guidelines would be potentially instrumental in supporting equitable healthcare delivery, enabling a more unified and effective response to global health challenges.

Together, these multi-level governance structures—combining institutional oversight, national standards, and international alignment—form a comprehensive framework that can guide the ethical, transparent, and equitable use of AI in pathology. Such a framework could help prevent misuse, protect patient rights, and ensure that AI advancements benefit healthcare systems universally rather than exacerbating existing disparities.

### Restricting algorithmic evolution: governing AI to preserve ethical and professional integrity

Another significant consideration in the governance of AI within pathology is the need to restrict unchecked algorithmic evolution. In *Nexus: A Brief History of Information Networks from the Stone Age to AI*, Harari highlights a critical juncture in technological development where self-evolving algorithms, if left unchecked, may prioritize efficiency and scalability over the ethical, interpretive, and contextual nuances essential to human-led systems.^75^ This observation is particularly relevant to pathology, where diagnostic processes inherently demand a combination of analytical precision, clinical judgment, and empathy—qualities that cannot be entirely replicated or governed by autonomous algorithms.

Harari's insights underscore the necessity of embedding robust oversight mechanisms into AI development to prevent its evolution into systems that operate independently of human expertise. Without such measures, there is a risk that AI in pathology could shift from a collaborative tool to an autonomous operator, potentially undermining professional roles, eroding public trust, and exposing healthcare systems to unforeseen risks.

To mitigate this, the pathology community must advocate for algorithmic constraints that preserve AI's augmentative role while preventing its progression into autonomous decision-making frameworks. Key strategies include mandating explainability in AI systems, requiring continual human oversight for critical diagnostic tasks, and instituting iterative validation processes to ensure alignment with clinical and ethical standards. Moreover, international collaboration on governance frameworks, drawing from precedents set by organizations like the WHO, could ensure consistency in AI oversight while accounting for regional differences in healthcare systems.

By addressing algorithmic evolution as a governance priority, the pathology community has an opportunity to shape the future trajectory of AI integration. This proactive stance will help safeguard the integrity of diagnostic practices, maintain the primacy of human expertise, and ensure that the benefits of AI are realized within a framework that upholds professional values and prioritizes patient care.

### Professional and public engagement

Engaging both the professional community and the public is essential in shaping a future where AI complements pathology.^5^ Transparent communication and educational initiatives can foster trust in AI-assisted diagnostics, especially as organizations work to demystify AI's role in health care.

Professional societies, such as the American Society for Clinical Pathology and the RCPath, have pioneered public engagement initiatives. American Society for Clinical Pathology 's *Artificial Intelligence is Here to Stay. Learn How it May Impact Patient Care* webinar provides education on how AI enhances, rather than replaces, the diagnostic work of pathologists.^122^ These programs break down complex AI processes through webinars, community workshops, and interactive demonstrations, ensuring that both patients and practitioners understand AI's supportive role in diagnostics.^122^

Similarly, RCPath's *Pathology Open Days* provides the public with a behind-the-scenes view of diagnostic processes, including AI applications in cancer detection and disease classification.^123^ These events underscore transparency, allowing attendees to see firsthand how AI is integrated into pathology workflows to augment human expertise.

Moving forward, expanding these initiatives through online platforms, collaborations with patient advocacy groups, and interactive Q&A sessions can further build public trust. By keeping the public informed, professional organizations reinforce AI as a tool that supports rather than competes with pathologists, laying a foundation of trust and transparency essential for AI's ongoing integration in pathology.

## Conclusion: augmenting pathology with AI—preserving the human touch

The integration of AI into pathology is not a narrative of certain replacement but one of potential augmentation. Drawing on historical parallels, such as the transition from analog to digital imaging in radiology, we can envision a future where AI enhances rather than eclipses the pathologist's role, preserving the essential human touch in the fabric of medical diagnostics. Just as digital imaging empowered radiologists by improving efficiency and accuracy yet relied on their expertise for interpretation and clinical judgment, AI in pathology has the potential to support and expand pathologists’ capabilities rather than replace them. This balanced integration between technology and human insight is essential for pathology's continued evolution and effectiveness.

Much like the introduction of microscopes, which revolutionized pathology by providing unparalleled detail yet required skilled interpretation, AI offers powerful analytics that may augment pathologists' insights, enhancing diagnostic accuracy. Similarly, AI functions like a global positioning system for drivers, offering valuable guidance and insights without removing the need for human decision-making and adaptation to clinical context. Artificial intelligence tools, like calculators for mathematicians, perform rapid calculations that support the diagnostic process while depending on the pathologist's foundational knowledge to interpret and contextualize findings, and akin to the shift from paper records to electronic health records, which streamlined workflows and data access for clinicians, AI too has the potential to bring efficiency without displacing the critical human oversight necessary for nuanced patient care.

To emphasize the value of this balanced approach to both clinical colleagues and administrators, the following points underscore the irreplaceable aspects of human expertise and the complementary advantages of AI augmentation:

• **Enhanced diagnostic accuracy:** AI has the capacity to support pathologists by identifying subtle patterns and anomalies that may be missed, potentially improving overall diagnostic accuracy in complex cases.
• **Streamlined workflows:** AI may reduce routine workloads, allowing pathologists to concentrate on cases that require nuanced interpretive skills, ultimately enhancing job satisfaction and productivity.
• **Consistency and quality across institutions:** AI has the potential to facilitate standardized, high-quality diagnostic practices across various healthcare settings, contributing to equitable patient care.
• **Critical oversight and interpretation:** Pathologists bring essential contextual knowledge to each case, interpreting findings within the broader clinical picture—an element AI alone cannot replicate.
• **Building trust through personalized patient interaction:** Maintaining the pathologist's role in patient care bridges the gap between technology and patient-centered practice, fostering trust and ensuring compassionate, personalized care.
• **Robust governance and human control over AI**: The integration of AI must be governed by clear and effective frameworks, ensuring strong human control over decision-making thresholds to prevent unintended consequences and safeguard the critical professional expertise of pathologists.

The future of AI in pathology lies in this harmonious blend of technology and human expertise, where AI has the potential to serve as a powerful tool that augments the pathologist's role. By preserving the indispensable human touch that remains at the core of effective and empathetic diagnostics, AI enhances pathology's capabilities without displacing the crucial interpretive, contextual, and compassionate elements that only a skilled pathologist can provide. Artificial intelligence can serve as a powerful, complementary tool that may empower pathologists by improving diagnostic accuracy, streamlining workflows, and reducing variability across institutions. Rather than diminishing the profession, AI can expand the pathologist's capabilities, enabling them to focus on complex cases that require in-depth interpretive skills and contextual understanding. This synergy may preserve the profession's integrity by reinforcing the pathologist's role as an essential decision-maker, especially in nuanced clinical scenarios where human expertise remains irreplaceable.

Looking forward, to ensure AI's continued positive impact in pathology, it is essential that pathologists take an active role in guiding its integration. Pathologist-led research and involvement in AI development will help ensure that the tools created align with clinical needs, ethical standards, and patient-centered care. As the field of AI in pathology evolves, it will be crucial to explore further integration methods that can enhance diagnostic accuracy and workflow efficiency without compromising the pathologist's central role. This requires a robust governance framework, with strong human control over AI decision-making thresholds, to avoid potential unintended consequences and safeguard the critical professional expertise of pathologists. Additionally, emerging AI tools, such as those capable of deep learning in personalized medicine, hold the potential to revolutionize the way pathologists approach complex diagnostic challenges. Continued research and innovation in these areas will be vital to help harness the full capabilities of AI, ensuring that its potential remains an augmentative tool that complements, rather than replaces, the expertise of the pathologist.

## Funding

The article-processing fee for this article was funded by an Open Access Award given by the Society of ‘67, which supports the mission of the Association for Academic Pathology to produce the next generation of outstanding investigators and educational scholars in the field of pathology. This award helps to promote the publication of high-quality original scholarship in *Academic Pathology* by authors at an early stage of academic development.

## Declaration of competing interest

The authors declare that they have no known competing financial interests or personal relationships that could have appeared to influence the work reported in this paper.
